# International comparison of observation-specific spatial buffers: maximizing the ability to estimate physical activity

**DOI:** 10.1186/s12942-017-0077-9

**Published:** 2017-01-23

**Authors:** Lawrence D. Frank, Eric H. Fox, Jared M. Ulmer, James E. Chapman, Suzanne E. Kershaw, James F. Sallis, Terry L. Conway, Ester Cerin, Kelli L. Cain, Marc A. Adams, Graham R. Smith, Erica Hinckson, Suzanne Mavoa, Lars B. Christiansen, Adriano Akira F. Hino, Adalberto A. S. Lopes, Jasper Schipperijn

**Affiliations:** 1 Health and Community Design Lab, Schools of Population and Public Health and Community and Regional Planning, University of British Columbia, Vancouver, BC Canada; 2 Urban Design 4 Health, Inc., Rochester, NY USA; 3 Department of Family and Preventive Medicine, University of California San Diego, San Diego, CA USA; 4 School of Public Health, The University of Hong Kong, Hong Kong, China; 5 Institute for Health and Ageing, Australian Catholic University, Melbourne, Australia; 6 Exercise Science and Health Promotion Program, School of Nutrition and Health Promotion, Global Institute of Sustainability, Arizona State University, Phoenix, AZ USA; 7 Institute for Environment, Sustainability and Regeneration, Staffordshire University, Stoke-on-Trent, UK; 8 Faculty of Health and Environmental Sciences, Auckland University of Technology, Auckland, New Zealand; 9 McCaughey VicHealth Community Wellbeing Unit, School of Population and Global Health, University of Melbourne, Melbourne, Australia; 10 SHORE and Whāriki Research Centre, School of Public Health, Massey University, Palmerston North, New Zealand; 11 Department of Sport Science and Clinical Biomechanics, University of Southern Denmark, Odense, Denmark; 12 Department of Physical Education, School of Life Science, Pontifícia Universidade Católica do Paraná, Curitiba, Brazil; 13 Department of Physical Education, Centro Universitario Campos de Andrade, Curitiba, Brazil

**Keywords:** Network buffer, Built environment, Reliability, Self-reported physical activity, GIS methods

## Abstract

**Background:**

Advancements in geographic information systems over the past two decades have increased the specificity by which an individual’s neighborhood environment may be spatially defined for physical activity and health research. This study investigated how different types of street network buffering methods compared in measuring a set of commonly used built environment measures (BEMs) and tested their performance on associations with physical activity outcomes.

**Methods:**

An internationally-developed set of objective BEMs using three different spatial buffering techniques were used to evaluate the relative differences in resulting explanatory power on self-reported physical activity outcomes. BEMs were developed in five countries using ‘sausage,’ ‘detailed-trimmed,’ and ‘detailed,’ network buffers at a distance of 1 km around participant household addresses (*n* = 5883).

**Results:**

BEM values were significantly different (*p* < 0.05) for 96% of sausage versus detailed-trimmed buffer comparisons and 89% of sausage versus detailed network buffer comparisons. Results showed that BEM coefficients in physical activity models did not differ significantly across buffering methods, and in most cases BEM associations with physical activity outcomes had the same level of statistical significance across buffer types. However, BEM coefficients differed in significance for 9% of the sausage versus detailed models, which may warrant further investigation.

**Conclusions:**

Results of this study inform the selection of spatial buffering methods to estimate physical activity outcomes using an internationally consistent set of BEMs. Using three different network-based buffering methods, the findings indicate significant variation among BEM values, however associations with physical activity outcomes were similar across each buffering technique. The study advances knowledge by presenting consistently assessed relationships between three different network buffer types and utilitarian travel, sedentary behavior, and leisure-oriented physical activity outcomes.

## Background

There is an increased interest worldwide in the impact of the built environment on physical activity and health-related outcomes. Numerous studies have reported positive associations between physical activity levels and measures of urban form including residential density, street connectivity, and land use mix [1–4]. Recent studies have further documented associations between built environment features and chronic disease outcomes [5, 6]. It is now thought that long-term impacts of transportation and land use decisions on health can be costly [7]. There is a growing awareness that changes to the built environment such as increased investment in transit, pedestrian and cycling infrastructures [8], and building more compact mixed use environments are required to reduce sedentary time [9, 10], promote physical activity [11], and stem escalating health care costs [12].

To quantify characteristics of the local built environment, researchers commonly collect spatial data and create built environment measures (BEMs) (e.g. intersections, transit stops, land use polygons) for an individual’s ‘neighborhood’ using geographic information systems (GIS) software. ‘Neighborhoods’ can be defined using the spatial distribution of locations near and associated with home, employment, or school. However, Census geography often forces an arbitrary depiction of a behavioural setting or neighbourhood for researchers and the inability to accurately capture how individuals conceptualize their neighborhood. This is known as the ‘modifiable areal unit problem’ (MAUP) and defined as issues of zone and scale arising from arbitrarily defined boundaries used to aggregate continuous spatial features [13, 14]. Kwan [15] argues that the ambiguity of the geographical context problem is due to the spatial and temporal uncertainty of where, when and how long individuals experience environmental influences. These issues can result in considerable mischaracterization of built environment exposure experienced by an individual. Mis-specification of the spatial neighborhood definition constitutes a violation of the ecological framework whose premise places the individual in the center of their environment [16].

An in-depth review by Brownson et al. [17] illustrates the lack of consistency in the field of physical activity research with respect to both the geographic units and scale used to define the spatial extent of an individual’s neighborhood. Several studies have shown that the choice of different geographic scales (e.g. 400 vs. 800 m buffers) used to create GIS-based BEMs has resulted in variation in the significance of associations between the built environment and physical activity and health outcomes [18–22]. Different spatial configurations (e.g. grids vs. buffers) at a consistent geographic scale have also been shown to produce varying results [18, 23]. Furthermore, algorithms used to create neighborhood buffers in Environmental Systems Research Institute (ESRI)’s ArcGIS software program have changed over time limiting comparative analyses with previous studies [24]. Variation in the geographic unit and scale used to characterize the built environment, combined with differences in software algorithms used to create neighborhood buffers, may be sufficient to result in inconsistent relationships between resulting BEMs and physical activity related outcomes. The methodological differences can mask consistencies that may actually exist between studies needed for policy makers to shift limited resources into investments that support active transportation.

**Fig. 1:**
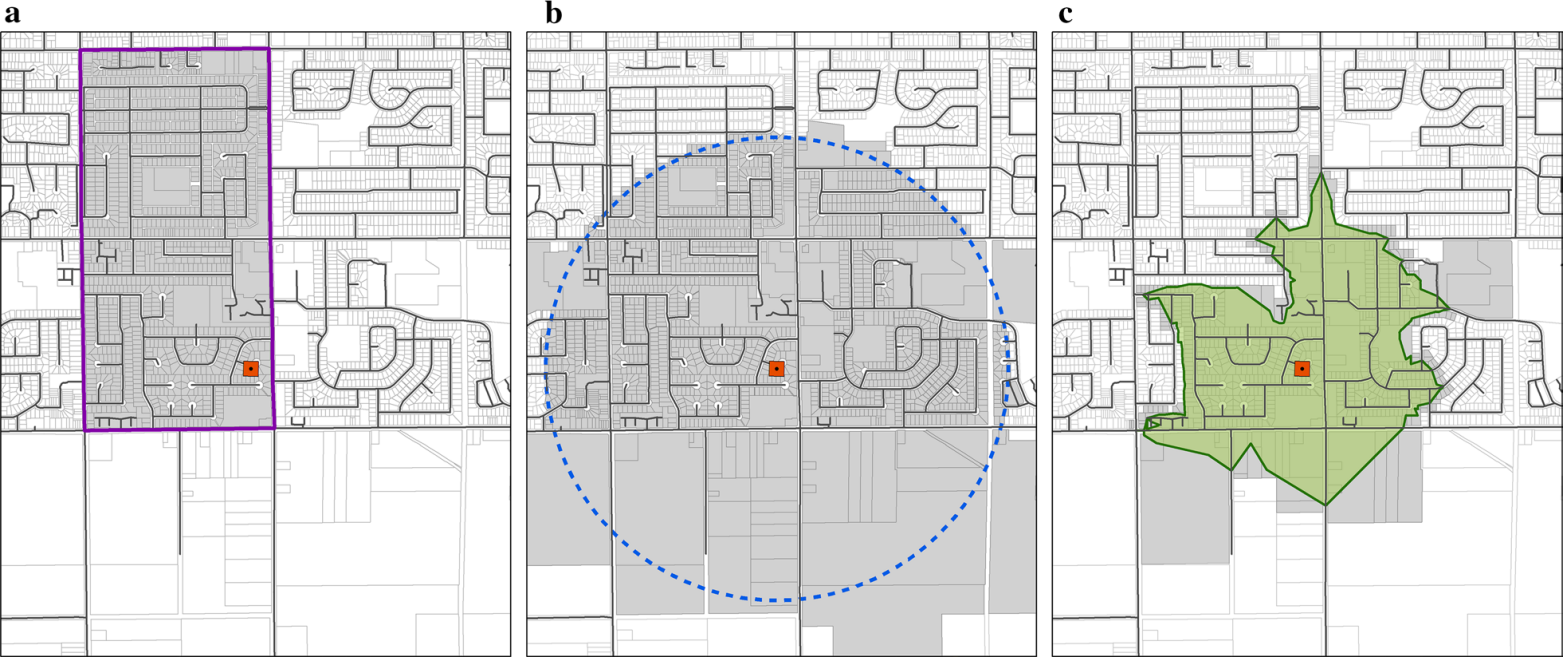
Land use parcels selected by three different neighborhood definition types. The *orange point* represents the participant’s home; the street network is highlighted in *black*; and *gray* polygons symbolize land use parcels that intersect each neighborhood type. **a** Census tract boundary, **b** 1 km circular (crow-fly) buffer, **c** 1 km street network buffer

Advancements in spatial analysis methods and software capabilities over the last two decades have advanced the approach used to model how an individual traverses their environment, reducing the impact of the MAUP. The earliest methods to define neighborhoods used pre-existing administrative units such as census tracts to assign an individual to a neighborhood (i.e. Fig. 1a); among the first of these studies was Frank and Pivo [25] who used census tracts to test associations between BEMs and travel behavior. Limitations to this approach include the potential inclusion of areas that are inaccessible on foot, and differences in built environment exposure for individuals who live near the edge of the spatial unit compared to the center.

The use of observation-specific circular (‘crow-fly’) buffers (i.e. Fig. 1b) offers improvements to the accuracy of the neighborhood definition by considering the individual’s actual location. This method has been applied in several studies [21, 26, 27], but this method does not consider how the street network allows or prevents access to specific locations within a given crow-fly or air-line distance.

The ability to create street network-based buffers using ESRI’s ArcView 3.3 software addressed this limitation and offered further refinement to the delineation of an individual’s neighborhood. This approach consists of creating ‘network buffer’ polygons at a given distance from the participant’s location based on the street network, better representing the area accessible to an individual. Network buffer polygons in ArcView 3.3 produced a ‘generalized’ buffer that could include areas not accessible to pedestrians from the street network. Later, ArcGIS 9.x offered the ability to create a more accurate, ‘detailed’ network buffer that followed the street network more closely (i.e. Fig. 1c). As described by Forsyth et al. [24], the progression from ‘generalized’ buffers in ArcView 3.3 to ‘detailed’ buffers in ArcGIS 9.x may limit the ability for results in the physical activity research field to be compared across time and between studies. Furthermore, depending on the parameters used to create ‘detailed’ buffers in Network Analyst (e.g. the ability to specify the perpendicular distance from the road centerline (“trim distance”) from which the polygon portion of the buffer is created), the resulting buffer area can be altered significantly. This methodological difference has the potential to affect comparability of findings between studies that use different types of ‘detailed’ network buffers.

More recently, the ‘sausage’ buffering method (also referred to as ‘line-based’ buffers) has been used by researchers as an alternate network-based buffering method [28–30]. The sausage buffering technique selects roads within a given distance of the participant, and creates a crow-fly buffer around the roads by a set distance (e.g. 25 m), thus selecting only the features that are directly accessible from the street network. Three strengths of the sausage buffer technique have been identified by Forsyth et al. [24]: (1) they are directly based on the pedestrian network where people travel, (2) they have similarities with other proprietary techniques, and (3) they can be reproduced using different GIS programs and software versions, allowing for stable, repeatable measurements to be produced across time.

A limited number of studies have compared whether BEMs and their associations with health-related outcomes differ when using circular buffers versus network buffers. Oliver et al. [31] was the first in the physical activity and health research field to compare differences in circular buffers versus network buffers in predicting physical activity, finding stronger associations between BEMs and walking behavior when using sausage network buffers. In a more recent study, James et al. [19] compared circular versus sausage buffers and found that only sausage buffers showed a statistically significant positive association between business count/density and walking for multiple buffer sizes.

Despite the utility of sausage buffers to produce consistent, repeatable buffers for quantifying the built environment accessible to the individual, limited sensitivity analyses have been conducted comparing the degree to which commonly used BEMs and their associations with physical activity outcomes may vary between sausage buffers versus other network buffers types derived using proprietary algorithms. Such comparisons are highly salient, as ESRI’s ArcGIS Network Analyst is a commonly used tool for generating neighborhood buffers. To the knowledge of the authors, Forsyth et al. [24] is the only study that systematically compared BEMs and health-related predictors produced by the sausage buffering technique to other network buffer types generated by ESRI’s ArcGIS Network Analyst tool (generalized, detailed, and detailed-trimmed). They found that measures of access to fast-food restaurants, convenience stores, and open space were highly correlated between buffer types (Pearson correlations > 0.94), while correlation coefficients for self-reported fast food purchases and fast-food restaurant counts, fruit and vegetable consumption and convenience store counts, moderate-and-vigorous physical activity (MVPA) and percent open space were similar in magnitude and statistical significance between buffer types, with the exception of open space and MVPA which showed slightly greater magnitude differences in correlation. Though this study is informative, it was conducted only in the US, and findings may vary in other countries that have very different built environment characteristics and physical activity patterns. Thus, it would be useful to compare the performance of various network buffering methods in an international study with greater diversity.

The present study seeks to build on previous research conducted by Forsyth et al. [24] by leveraging a large dataset of internationally developed, consistent built environment variables and commonly used physical activity outcomes across several countries. Each of the study areas exhibits different levels of urbanization and cultural backgrounds and supports testing relative differences in explanatory power of three different network buffering methods to predict physical activity outcomes.

The overall purpose of this study is to investigate whether different buffering techniques alter the predictive strength of BEMs on physical activity and sedentary behavior. Specifically, we aim to apply inferential modeling techniques to examine statistical differences among an expanded set of common BEMs calculated for detailed, detailed-trimmed, and sausage buffers around International Physical Activity and Environment Network (IPEN) Adult participants’ household addresses, and to assess whether the statistical relationship between various physical activity domains and BEMs calculated using the sausage buffer technique are significantly different compared to other network buffer techniques.

## Methods

### Participants

This study used cross-sectional built environment and physical activity data collected as part of the IPEN Adult study. The details of the study design have been published elsewhere [32, 33]. Briefly, an international study was conducted in 17 cities across 12 countries for the purpose of increasing intra-regional and inter-country comparability using a common research design and methodology, with the aim of ensuring a broad range of built environment features and use of comparable objective and self-reported measures of physical activity and the built environment. The overarching goal of the study was to inform evidence-based physical activity policies and interventions at both the international and country level to mitigate obesity and other chronic diseases.

For this study, data from a subset of the IPEN Adult study sites from five countries (Brazil, Denmark, New Zealand, United Kingdom, and the United States) were used based on each study site’s access to common spatial data to develop comparable built environment measures, availability of internal GIS expertise to produce the required variables and willingness to participate in the spatial buffer comparison analysis. The home is a common and widely investigated location for quantifying built environment exposure within the literature among similar physical activity studies and was selected as the most suitable representation of participant neighborhood environment for comparability across the five countries [34]. The combined dataset from these sites consists of 5883 adults from five countries (Brazil, Denmark, New Zealand, United Kingdom, and the United States) Participant recruitment at each study site was stratified by socio-economic status (SES) and transport-related walkability, which have been described in detail in other publications [32].

### Buffer development

Three different network buffer types were used to compare differences in BEM relationships with physical activity outcomes: (1) detailed; (2) detailed-trimmed; and (3) sausage. The buffers were used to identify all spatial features that are accessible within 1 km (10–15 min walk) of each participant’s home, a distance commonly used to typify the environment accessible within reasonable walking distance [17, 35]. The buffers were generated in GIS based on a walkable street network derived from the road class type that excluded limited access highways and highway entrance ramps where pedestrians were not permitted to traverse. For the detailed-trimmed and the sausage buffers, trim distances of 25 and 75 m were produced to compare and analyze two threshold distances from the road network balancing the need to include adjacent polygon features with further set back from the roadway, while not erroneously including others that cannot be accessed or are on adjacent streets beyond 1 km at the edges of buffers.

**Fig. 2:**
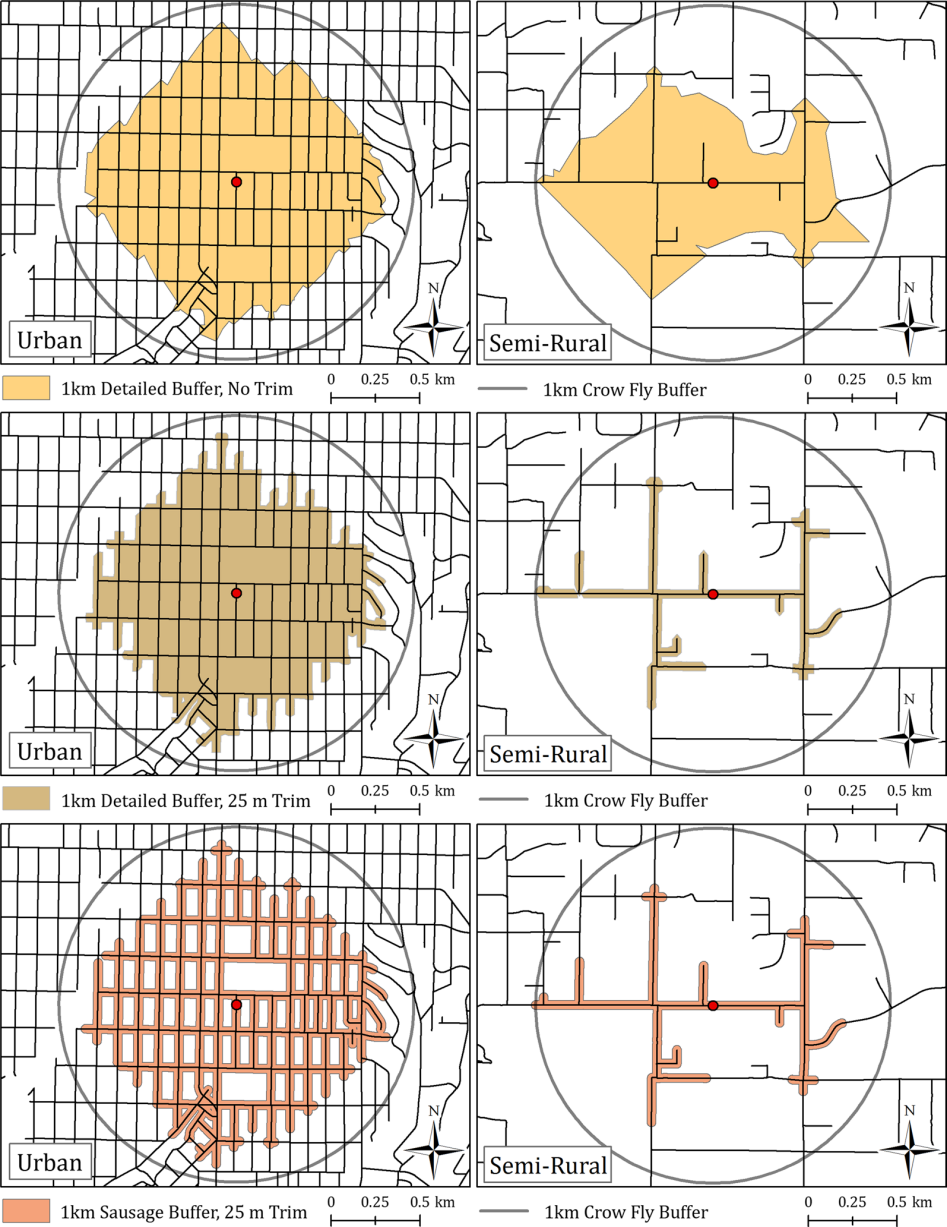
Illustration of three network buffer types that were generated around participant households. (1) Detailed buffer; (2) Detailed buffer trimmed on either side of the street network to 25 m; (3) Sausage buffer with 25 m radius on either side of street

All three types of buffers compared for this study were generated using the ESRI ArcGIS 10.1 software and the Service Area Solver within the Network Analyst extension. The ‘detailed’ polygon generation option was enabled to generate network polygons for both ‘detailed’ and ‘detailed-trimmed’ buffers and trim distances of 25 and 75 m was applied to the latter. Sausage buffers were created following methods described in detail elsewhere [36]. The Service Area Solver was used to generate lines along the road network to a distance of 1 km using the line generation dialog. Output line features were then dissolved based on participant identification number to obtain one set of line features per participant; the line features were then buffered by 25 and 75 m to complete the sausage buffer polygons. Figure 2 depicts the three different buffer types in urban and semi-rural environments.

### Dependent variables

Five self-reported physical activity outcomes from the IPEN Adult study were used to test sensitivity of BEMs on physical activity between the three buffer types: walking for transportation (days/past week); time spent walking for transportation (minutes/past week); walking for leisure (days/past week); time spent walking for leisure (minutes/past week) and; time spent sitting (minutes/past week). All outcome measures are items from the International Physical Activity Questionnaire (IPAQ; long version). The full IPAQ was originally developed and evaluated in 12 countries on five continents and found to have good test–retest reliability (ICC = 0.46–0.96) and fair-to-moderate criterion validity (median ρ = 0.30) compared against accelerometer measures [37]. In this study, outcome measures for both walking for transportation and walking for leisure during the past 7 days used IPAQ items on walking ‘frequency’ (days per week) and ‘duration’ (computed from ‘days per week’ times ‘typical minutes per day’). Total time spent sitting during the last 7 days was based on two other IPAQ items and computed as a weighted sum of minutes spent sitting during week days (times 5) plus minutes spent sitting during weekend days (times 2).

### Independent variables

Eleven BEMs were derived in GIS at the 1 km buffer level for (1) detailed buffers, (2) detailed-trimmed buffers, and (3) sausage buffers. The methods for computing and ensuring comparability of GIS variables in the IPEN Adult study have been previously described [38]. The following BEMs were calculated, all of which are commonly used in physical activity and health research: single and multi-family net residential density (units per residential km^2^), road intersection density, bus and rail stop access (count and density), private recreation access (count and density), public park access (count and total acres), land use mix (an entropy equation calculating evenness of residential, retail (including retail/commercial, entertainment and food-related) institutional/civic, and recreational land areas on a scale of 0–1), and an overall walkability index adapted from Frank et al. [39] (sum of z-scores of net residential density, land use mix, and intersection density). The input data used to create these measures are described in further detail elsewhere [38]. Polygon features (e.g. parks) were assigned to buffers if any portion of the polygon geometry intersected the buffer. Point features (e.g. transit stops, private recreation locations) were moved (‘snapped’) to the closest point on the road network before aggregating to buffers to ensure that all destinations located near the street network were accurately acquired by the buffers. BEMs were generated for both snapped and unsnapped point features. Differences in resulting BEMs coefficients were compared, with no statistically significant differences found between coefficients resulting from the two methods. BEM values based on the snapped point features method are reported here.

Individual socio-demographic covariates used in the analyses were derived by questionnaire and included age, gender, education level (less 12 years/high school, high school degree or some college, or university degree or higher), marital status (married/living with partner or other), and employment status (yes/no). The participant’s SES and walkability categories were included as covariates in the modeling process. In addition, the cluster unit (census-based administrative unit) the participant was recruited from was included as a random effect in the modeling process.

### Statistical approach

All analyses were conducted for a single, pooled data set as well as for the following strata:

1. *City:* Five countries provided data for eight different cities as follows: Curitiba, Brazil (BR); Aarhus, Denmark (DK); North Shore (NZ-NS), Wellington (NZ-WE), and Christchurch (NZ-CC), New Zealand; Stoke-on-Trent, United Kingdom (UK); and Seattle/King County (US-KC) and Baltimore (US-MD), United States. Analyses were repeated after stratifying the pooled data set by study city, resulting in eight sets of city-level results.
2. *Neighborhood SES/walkability quadrant:* (1) Analyses were repeated after stratifying the pooled data set by SES/walkability quadrant, resulting in four sets of quadrant-level results, and (2) stratifying the city-level data set by SES/walkability quadrant, resulting in four additional sets of quadrant-level results for each of the eight cities.

First, a descriptive analysis of BEMs calculated for each buffer type was conducted using both the full sample of data (pooled across all study sites) and stratified by quadrants (low/high walkability by low/high SES) and cities. BEMs calculated for sausage buffers were compared to BEMs for (1) detailed-trimmed buffers and (2) detailed buffers by *t* test and by mean squared error.

Next, using only the pooled data, generalized additive mixed models (GAMMs) were used to examine shape (linear vs. curvilinear), strength, and direction of associations between outcomes and BEMs created using each buffer method [40, 41]. GAMMs provide two principal advantages for this analysis: (1) the shape of the relationship between outcomes and BEMs does not need to be pre-determined, rather the shape is derived from the data, and (2) the GAMMs are able to model both fixed and random effects, and thus are appropriate for modeling hierarchical data (i.e. participants nested within neighborhoods nested within cities).

BEM regression coefficients calculated for each sausage buffer model were compared to the equivalent coefficients calculated for the detailed-trimmed and detailed buffer models in terms of absolute differences in coefficient and *p* value and as evaluated by *z* test. BEMs were standardized (using z-scores) to allow for easier comparison between coefficient values. The moderating impact of study site on the BEM coefficients was also tested by developing separate study site-specific models where needed. All models adjusted for the socio-demographic covariates listed above. Although 25 and 75 m trim distances were tested for sausage buffer models, only results from 25 m trim sausage buffers are reported in the following results due to the similarity in results yielding no statistically significant differences between the two trim distances. All model analyses were performed in R (R Development Core Team, 2014) using the packages ‘foreign’, ‘mgcv’, ‘metrics’ and ‘nlme’ [42–45].

## Results

The study sample was comprised of 5883 adult participants aged 18–66 years. Table 1 provides a summary of the mean socio-demographic characteristics for each city. Education level displayed some of the largest variability among demographic variables (e.g. ‘less than high school’ ranging from 0.8% in NZ-WE and 1.3% in US-KC to 33.8% in UK and 28.8% in BR).

**Table 1:** Mean socio-demographic characteristics by study site

Country	City	N	Age (SD)	% female	Education level			% Married	% Employed
					Less than high school (%)	High school/some college (%)	College graduate or higher (%)		
Pooled		*5883*	42.4 (12.4)	53.0	10.9	42.2	46.3	59.2	77.9
United States (US)	Seattle (KC)	1287	44.0 (11.0)	45.2	1.3	35.4	63.0	63.2	81.3
	Baltimore (MD)	912	46.6 (10.7)	52.3	2.0	30.3	67.2	60.5	82.6
New Zealand (NZ)	North Shore (NS)	511	40.9 (11.8)	63.9	3.7	57.3	38.0	70.4	77.7
	Wellington (WE)	496	39.2 (12.6)	51.2	0.8	47.0	52.2	56.7	86.7
	Christchurch (CC)	495	41.7 (12.6)	55.8	10.7	57.2	31.9	55.4	79.6
Denmark (DK)	Aarhus	642	38.9 (13.9)	56.7	7.3	43.1	46.6	65.4	74.6
Brazil (BR)	Curitiba	697	41.1 (13.2)	52.9	28.8	32.4	38.7	58.1	77.6
United Kingdom (UK)	Stoke-on-Trent	843	43.0 (13.3)	56.1	33.8	51.7	14.0	44.8	64.4

Walking for transportation frequency varied by country, with participants in NZ-WE walking most often (4.1 days/week), followed by BR (3.5 days/week) (Table 2). Walking for leisure was engaged in most frequently by DK (2.8 days/week) and NZ-WE (2.3 days/week) participants. Time spent sitting was highest in DK (2676 min/week) followed by the US-KC and US-MD study sites, averaging 2555 and 2545 min/week respectively.

**Table 2:** Mean physical activity outcomes by study site

Study site	N	# of days walking for transport (days/past week)	Time spent walking for transport (min/past week)	# of days walking for leisure (days/past week)	Time spent walking for leisure (min/past week)	Time spent sitting (min/past week)
Pooled	5883	3.0	164.1	1.9	112.1	2396
US-KC	1287	2.9	173.9	2.1	120.4	2555
US-MD	912	2.9	171.4	2.0	104.8	2545
NZ-NS	511	2.5	86.1	1.7	84.4	2402
NZ-WE	496	4.1	180	2.3	112.4	2488
NZ-CC	495	2.0	79.7	1.5	75.2	2296
DK	642	3.3	190.8	2.8	198.7	2676
BR	697	3.5	153.3	1.2	54.3	1980
UK	843	3.0	218.4	1.6	129.3	2116

### Buffer comparison

**Fig. 3:**
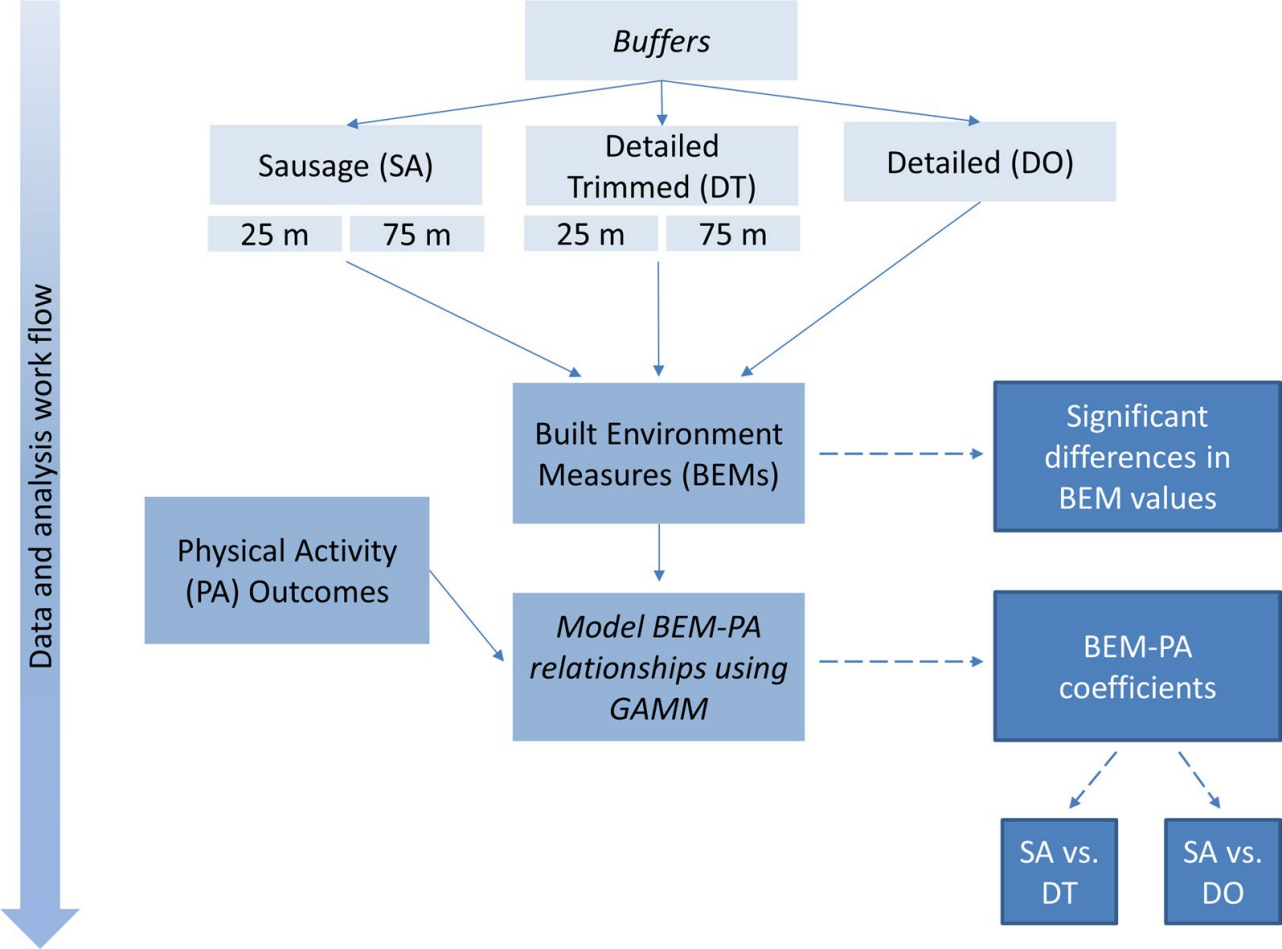
Analysis workflow process comparing BEMs from three source network buffering types with both 25 and 75 m trim distances for sausage (SA) and detailed trimmed (DT) with physical activity (PA) outcomes. BEM-PA relationships are modeled using generalized additive mixed models (GAMM) to determine statistically significant pairs

Across all study sites, sausage buffers had the smallest average area (0.74 km^2^). The detailed-trimmed buffers had an average area of 0.89 km^2^, while detailed buffers covered the largest area (1.29 km^2^). Ninety pairs of BEMs developed through the three network buffering techniques were compared across the eight study cities. Figure 3 conveys the sequence of steps used to compare BEMs from the three buffering types with the physical activity health outcomes. In 96% of cases, sausage buffer BEM values were significantly different from detailed-trimmed buffer BEMs values, while in 89% of cases, sausage buffer BEM values were significantly different from detailed buffer BEMs values. Density-related BEMs tended to show more differences between buffer types than count-based BEMs, presumably because the buffer area can vary greatly by buffer type.

All possible combinations (*n* = 450) of built environment-physical activity pairs for pooled and city-specific analyses were run for (1) sausage versus detailed-trimmed buffers and (2) sausage versus detailed buffers. No statistically significant differences in associations of sausage versus detailed-trimmed buffer BEM with physical activity outcomes were found. The *p* value for BEM coefficient associations with outcome variables differed in significance level (i.e. the coefficient for only one buffer type had a *p* < 0.05) in 17 cases (4%), with the sausage buffer BEM statistically significant in 5 cases, and the detailed-trimmed buffer BEM statistically significant in 12 cases. The t-statistic was higher for the sausage buffer BEM coefficient in 45% of cases and higher for the detailed-trimmed buffer BEM coefficient in 55% of cases.

When comparing sausage buffers with detailed buffers, the *p* value for BEM coefficient associations with outcome variables differed in significance (*p* < 0.05) in 40 cases (9%), with only the sausage buffer BEM significant in 25 cases and only the detailed buffer BEM significant in 15 cases. The t-statistic was higher for the sausage buffer BEM coefficient in 48% of cases and higher for the detailed buffer BEM coefficient in 52% of cases.

It was also observed that BEMs were much more likely to be significantly associated with the transportation outcomes rather than the leisure physical activity or sitting outcomes. For one or both buffer types, the BEM was significantly associated with the outcome in:

- Sausage versus detailed-trimmed buffers: 41% of transportation walking models; 11% of leisure physical activity models; 7% of sedentary behavior models;
- Sausage versus detailed buffers: 44% of transportation walking models; 13% of leisure physical activity models; 9% of sedentary behavior models.

For both the sausage versus detailed-trimmed and sausage versus detailed buffer comparisons, the table below summarizes the number of pairs of BEM coefficients differing in statistical significance (the coefficient for only one buffer type had a *p* < 0.05) for the pooled and city-level analyses (Table 3). For both the sausage versus detailed-trimmed and sausage versus detailed buffer comparisons, the most statistically significant BEM coefficient pairs were found for the pooled city analysis (primarily due to having the largest sample size and statistical power), followed by US-KC and US-MD. The most BEMs coefficient pairs differing in statistical significance were found for US-KC and US-MD.

**Table 3:** Comparison of BEM-physical activity (PA) coefficients: statistically significant pairs, by study site

Study site	*N*	# of pairs of significant BEM-PA coefficients*		# of pairs differing in BEM-PA statistical significance*	
		SA-DT	SA-DO	SA-DT	SA-DO
Pooled	5883	26	24	2	2
USA- KC	1287	24	23	4	4
USA-MD	912	11	11	4	1
NZ-NS	511	4	2	0	0
NZ-WE	496	6	3	1	1
NZ-CC	495	2	0	2	4
DK	642	6	5	2	2
BR**	697	2	2	1	0
UK***	843	2	2	1	0

*SA*-*DT* sausage buffer versus detailed-trimmed buffer, *SA*-*DO* sausage buffer versus detailed buffer

* Out of 55 comparisons per country/city; ** BR only has 35 comparisons; *** UK only has 30 comparisons

Across all BEM coefficients, the count of *statistically significant coefficient pairs* for both sausage versus detailed-trimmed and sausage versus detailed comparisons was highest for the transportation walking duration outcome, followed by the transportation walking frequency outcome (Table 4). Across all outcomes, the count of *statistically significant BEM coefficient pairs* was highest for net residential density and transit stop count measures for both sausage versus detailed-trimmed and sausage versus detailed buffer comparisons.

**Table 4:** Comparison of BEM model coefficients with physical activity (PA) outcomes

	(1) # of pairs of significant (*p* < 0.05) BEM-PA coefficients*										(2) # of pairs *differing* in BEM-PA coefficient significance (*p* < 0.05)*									
	# of days walking for transport (days/past week)		Time spent walking for transport (min/past week)		# of days walking for leisure (days/past week)		Time spent walking for leisure (min/past week)		Time spent sitting (min/past week)		# of days walking for transport (days/past week)		Time spent walking for transport (min/past week)		# of days walking for leisure (days/past week)		Time spent walking for leisure (min/past week)		Time spent sitting (min/past week)	
Buffer comparison	SA-DT	SA-DO	SA-DT	SA-DO	SA-DT	SA-DO	SA-DT	SA-DO	SA-DT	SA-DO	SA-DT	SA-DO	SA-DT	SA-DO	SA-DT	SA-DO	SA-DT	SA-DO	SA-DT	SA-DO
Overall public park land area sum, m^2^***	0	0	1	1	0	0	0	0	0	0	2	1	0	0	0	0	0	0	0	0
Overall public park count, m^2^***	2	2	2	2	0	0	0	0	0	0	0	0	0	0	0	1	0	0	0	0
Intersection count	2	2	5	5	0	0	0	0	0	0	0	1	0	1	0	0	0	0	0	0
Intersection density (intersections/km^2^)	1	2	2	3	0	0	0	0	0	0	2	1	1	2	0	2	1	2	1	1
Residential, land area sum, net density***	5	5	4	3	2	0	4	2	0	0	0	0	0	2	0	2	0	2	0	1
Bus/rail stop count	4	4	3	3	1	1	1	1	2	2	2	1	2	2	0	0	0	1	0	0
Bus/rail stop density (stops/km^2^)	2	2	3	2	0	0	1	0	2	2	1	1	0	2	0	0	0	0	0	0
Private recreation count	3	3	4	4	1	1	0	0	0	0	0	0	0	0	0	0	1	1	0	0
Private recreation density (count/km^2^)	3	3	4	4	1	1	1	0	0	0	0	0	0	0	1	0	1	3	0	0
Mixed use**	3	2	3	3	1	0	1	0	0	0	1	1	0	1	0	1	0	1	0	1
Walk index***	4	3	3	3	1	0	1	1	0	0	0	2	0	1	0	1	0	0	1	1
Total # pairs	29	28	34	33	7	3	9	4	4	4	8	8	3	11	1	7	3	10	2	4

(1) Statistically significant pairs; (2) Pairs *differing* in statistical significance

*SA*-*DT* sausage buffer versus detailed-trimmed buffer, *SA*-*DO* sausage buffer versus detailed buffer

* Out of 9 comparisons/BEMs; ** only 8 comparisons available; *** only 7 comparisons available

The count of BEM-physical activity coefficient pairs *differing in statistical significance* was highest for the transportation walking frequency outcome for the sausage versus detailed-trimmed comparison (Table 4). Across all outcomes, BEM coefficient pairs differing in statistical significance were most likely for intersection density and transit stop count BEMs. For the sausage versus detailed comparison, BEM coefficient pairs *differing in statistical significance* were most likely for the transportation walking duration outcome, followed by the leisure walking duration outcome. Across all outcomes, BEM coefficient pairs differing in statistical significance were most likely for intersection density and residential density measures.

## Discussion

The aim of this study was to investigate how three types of network buffers differed in measuring a set of commonly used BEMs, and to test their relative ability to predict physical activity outcomes. Defining an individual’s neighbourhood using network buffers has become a commonly applied practice in public health research due to increased spatial accuracy in capturing an individual’s exposure to urban form features compared to coarser spatial units such as pre-defined administrative areas or circular buffers. However, differences in the techniques available to generate network buffers both within and between GIS platforms are a limiting factor in the ability to compare results between studies. Comparability is further hindered by methodological differences in the GIS software used impacting the network buffering process.

Results from this international study of cities with diverse built environments indicated that BEM values often differed significantly by buffer type employed. However, in the vast majority of cases, BEM associations with each physical activity outcome yielded a similar level of statistical significance for the sausage buffering technique as the detailed and detailed-trimmed network buffers derived using proprietary algorithms. However, for 9% of sausage versus detailed buffer model pairs, BEM coefficient associations with outcome variables differed in significance. This discrepancy warrants further investigation, and may be due to larger variation in buffer area between sausage and detailed buffers and subsequent impact on density-related BEMs.

Despite the similarity of the ability to predict physical activity among the various types of buffers examined, the findings of this study yield analogous conclusions to those provided by Forsyth et al. [24] using a smaller set of BEMs, that the sausage method remains the most defensible method for creating network buffers due to repeatability and consistency in buffer shape across GIS platforms. Specifically, the sausage buffering approach produces a representative area for area-based measures regardless of street network connectivity, and ensures that only point, line and polygon features that are accessible from the road network are used to quantify the built environment. However, it should be noted that variation in the spatial representation of urban form features (e.g. parcels may be in centroid or polygon format) used to create BEMs may yield inconsistencies between studies, despite the use of a uniform buffering approach.

The present study used a 25 m trim buffer along the street network for the detailed-trimmed and sausage buffers, with point locations snapped to the road network to ensure that all features were captured by the buffer. The buffer distance from the road influences the number of point and polygon features captured by the buffer; distances that are too large could result in features that are not accessible from the road network being included, while buffer distances that are too small may result in features that are accessible being excluded. Road network data are typically available as centreline features, thus a minimum buffer distance of 25 m from the road network is suggested when creating network-based buffers, due to variability in road and sidewalk width depending on geographic context. Other studies have used larger trim distances ranging from 50 [19, 29, 31, 46] to 150 m [24] to capture point features that are not snapped to the road network.

Strengths of this study included the diversity of urban form from which BEMs were collected (five countries), allowing the ability to test the validity and transferability of the sausage buffering technique across a wide range of environment types. Extensive procedures were implemented to maximize the comparability of BEMs across cities [38]. This study uses a large set of BEMs commonly used in health research, allowing for an in-depth comparison of how different BEMs and associations with physical activity outcomes specific to purpose (transport or leisure) vary across buffer type. Another strength of the current study is the comparison of BEMs produced by the different buffers using inferential modeling techniques.

Weaknesses of this study include lack of availability of pedestrian features in the road networks used in this global investigation (such as non-motorized trails, or other pedestrian only ways which do not follow the road network) to create buffers. The road only buffers may be smaller than buffers created using roads with pedestrian connections. Road network data that include pedestrian pathways have limited availability, are often prohibitively expensive to create or are developed for the purpose of a tracking inventory rather than network analysis resulting in spatial connectivity issues. In the case of this study, a road network containing non-motorized pedestrian trails was only readily available for one study regions. Another limitation is the lack of objective data collection including Global Positioning Systems (GPS) on study participants. Finally, the cross-sectional nature of the study design limited the causal interpretation of any findings.

A growing body of research suggests that place-based definitions focused on the residential neighborhood do not adequately capture the fact that daily activities are often conducted in multiple environments [15, 47]. Many studies have addressed the spatial and temporal variation in human activities through the collection of GPS data to create individual ‘activity spaces’ [46–48], allowing for increased understanding of contextual influences on physical activity. Alternative buffer methods based on GPS data (e.g. ellipse-shaped polygons, daily path areas) offer potential to redefine how an individual’s neighborhood is conceptualized. The resources and costs of implementing a participant GPS data acquisition often make it impractical for large epidemiological studies; especially those that are global and operating with many study regions. GPS provide a valuable method to objectively measure where people go and duration of time and engagement in specific activities and exposure to environmental phenomena. However, GPS does not have the ability to supplant the need for GIS-based methods to independently inventory and measure built environment features. Development of suitable buffers around key habitual locations, such as the home, will remain important to understanding the opportunities for behavior an environment offers. Future research investigating how street network-based buffer methods compare to alternative activity spaces definitions, such as those defined using GPS technology, will continue to advance understanding of built environment relationships with physical activity behaviors.

## Conclusions

To the knowledge of the authors, this study is the first to evaluate alternative forms of network buffers for capturing built environment attributes across multiple international study sites, demonstrating strong generalizability within a global context. The current study further advances the existing scholarship base by presenting consistent relationships between three different network buffer types for utilitarian transportation, sedentary behavior, and leisure-oriented physical activity outcomes. Further studies should compare the strength of BEM associations between different types of network buffers with other health-related measures such as objectively-measured physical activity, obesity and chronic health outcomes. Investigation into the use of these buffering methods may be applied to quantify exposure to other environmental phenomena, especially when data containing duration of exposure over time is available. Additional research may also seek to test the strength of BEM associations between different buffering techniques on specific population cohorts including those varying by age, gender, and income.

## Abbreviations

BEM: built environment measure
GAMM: generalized additive mixed model
GIS: geographic information systems
GPS: global positioning system
MVPA: moderate-and-vigorous physical activity
IPEN: International Physical Activity and the Environment Network
IPAQ: International Physical Activity Questionnaire
SES: socio-economic status
